# The effect of pre-habilitation programs on surgical outcomes among patients undergoing elective cardiac surgery

**DOI:** 10.6026/9732063002001935

**Published:** 2024-12-31

**Authors:** Hariprasad Gnanavelu, Kaushik Rajavel, Disha Tyagi

**Affiliations:** 1 Department of General Surgery, Bangalore Baptist Hospital, Bangalore, Karnataka, India; 2 Department of Orthopaedics, Madras Medical College, Chennai, Tamil Nadu, India; 3 Department of Internal Medicine, Deen Dayal Upadhyay Hospital, New Delhi, India

**Keywords:** Prehabilitation, cardiac surgery, postoperative complications, functional recovery, elective surgery, surgical outcomes

## Abstract

Pre-habilitation programs, designed to optimize physical, nutritional and psychological health before surgery, show promise in improving outcomes for elective cardiac surgery. This randomized controlled trial assessed the impact of a 4-week prehabilitation program on 100 patients undergoing elective cardiac surgery, comparing it to standard preoperative care. The prehabilitation group experienced significantly fewer postoperative complications (12% vs. 28%, p = 0.016), shorter hospital stays (6.4 ± 1.8 days vs. 8.2 ± 2.1 days, p < 0.001) and improved 30-day functional recovery (p = 0.005). These findings demonstrate that prehabilitation enhances recovery, reduces complications and shortens hospitalization, supporting its inclusion in preoperative protocols for elective cardiac surgery.

## Background:

The major risks associated with elective cardiac surgery are postoperative complications, longer recovery and significant functional limitation after the surgery [1]. The traditional preoperative care primarily focuses on assessing the surgical risk and optimizing medical conditions. However, the present approach often fails to use such an opportunity to make the patient more resilient, either physically or mentally, before surgery [2]. Targeted interventions performed before surgery to optimize a patient's condition emerged as a potentially promising approach to improve recovery and reduce complications, prehabilitation [3]. Three major broad categories of prehabilitation programs comprise physical exercise that enhances cardiovascular fitness, nutritional optimization in healing and psychological support to minimize anxiety and stress induced by surgery [4]. In this aim, the intention is towards enhancing a patient's general fitness in a total sense for better tolerance towards surgical stress and faster recovery [5]. Prehabilitation was found to reduce complications and improve outcomes in orthopedic and abdominal surgeries, but it has not been fully explored in the case of cardiac surgery [6]. This article will evaluate prehabilitation effects on the outcomes of surgical surgery on patients who are scheduled to undergo elective cardiac surgery. Therefore, it is of interest to understand whether it is possible for patients to recover faster as a result of prehabilitation and consequently, medical costs on the side of the patient increase by means of a longer stay in the hospital and complications [7].

## Methodology:

This was a randomized controlled trial conducted from January 2022 through December 2023. It involved selected elective cases of 100 patients assigned for CABG and valve replacement surgeries.

## Inclusion criteria:

[1] Patients aged between 50 and 80 years who are scheduled for elective cardiac surgery.

[2] Patients who do not suffer from severe comorbidities that will limit their participation in physical exercise.

## Exclusion criteria:

[1] Patients requiring emergency cardiac surgery.

[2] Patients with severe heart failure or other conditions contraindicated for physical activity.

## Study design:

Patients were randomly assigned to one of two groups:

[1] Group A: This was the prehabilitation group. There was a 4-week prehabilitation program of supervised physical exercises and nutritional counselling that went along with psychological support before surgery.

[2] Group B: The group which was put as control. The patients received standard preoperative care without any type of prehabilitation intervention.

## Prehabilitation program:

The three-part prehabilitation program involved.

[1] Physical exercise: Patients were subjected to aerobic and resistance training exercises which suited their fitness level. Sessions were conducted thrice a week with the aid of a physiotherapist.

[2] Nutritional optimization: Individualized nutritional counselling was provided to the patients to ensure adequate protein and calories intake before surgery in order to heal and recover well.

[3] Psychological support: Patients underwent relaxation techniques and the counseling sessions were focused on the decrease in preoperative anxiety level and increase in mental toughness before surgery.

## Data collection:

[1] Post-operative complications: Complications that a rose post-surgery including infections, respiratory disease and cardiac cases were documented.

[2] Functional recovery: Functional recovery was determined by the 6 minute walk test and Karnofsky Performance Status scale on day 30 of post-surgery.

[3] Duration of hospital stay: The number of days stayed in the hospital after surgery was recorded for all patients.

## Statistical analysis:

SPSS software version 26 was used to analyze the data. Continuous variables were presented as mean ± SD, whereas categorical variables were presented as percentages. Chi-square and t-tests were used to compare outcomes between groups; a p-value of less than 0.05 was regarded as statistically significant.

## Results:

Total of 100 patients was accrued. One group received the prehabilitation program in 50 patients and the remaining 50 patients received standard preoperative care. The following tables summarize the results in terms of postoperative complications, functional recovery and the length of hospital stay. Baseline characteristics in the two groups were well matched, which ruled out demographic confounding factors in the outcomes (Table 1). The prehabilitation group had significantly fewer postoperative complications than the control group, with reduced infections and pulmonary complications (Table 2). Patients in the prehabilitation group had much better functional recovery at 30 days post-surgery as shown by larger distances walked in the 6-minute walk test (Table 3). The Karnofsky Performance Status scale showed better scores for functional recovery at 30 days in the group of patients who had prehabilitation compared to the control (Table 4). Patients with prehabilitation were found to have shorter hospital stays than the control patients with statically significant differences (Table 5). The readmission rates of patients in the prehabilitation arm were lower but this was not statistically significant (Table 6). Patients who reported being satisfied with their care before surgery were more commonly found in the prehabilitation group (Table 7). The postoperative scores for pain were significantly lower in the prehabilitation group compared to the control at 24 and 48 hours post-surgery (Table 8). Patients who had prehabilitation extubated earlier than control; this is an indication that recovery was faster within the immediate postoperative period (Table 9). Patients with the prehabilitation group spent lesser time in the ICU in comparison to the control (Table 10).

## Discussion:

Preoperative programs significantly enhance outcomes after surgery for patients receiving elective cardiac surgery [8, 9]. Significantly less incidence of complications post-operatively, especially infections and respiratory, was reported among those patients who were assigned into the structured preoperative rehabilitation program compared to those of the standard preoperative care [10]. The results also go with the previous studies wherein a positive effect on benefits achieved in prehabilitation brings on a reduction in the development of complications during and following the surgical process [11, 12]. Except for reducing complications, prehabilitation also enhanced recovery in functional status through its better outcomes in the walk test 6 minutes after an exercise test and higher scores in the Karnofsky Performance Status, 30 days after an operation [13, 14]. That is why this might result from physical conditioning and heightened psychological toughness that a patient gains along the way when undergoing prehabilitation sessions [15]. Additional testimony to quicker recovery is hospital and ICU stay duration by patients in the prehabilitation groups [16, 17]. Interestingly, patients in the prehabilitation group also showed lower postoperative pain scores and a shorter time course to extubation, which suggests there are aspects of prehabilitation that improve physical recovery and the management of pain and comfort during the postoperative period [18, 19]. These benefits would be the combined effects of the physical and psychological component of the prehabilitation program preparing patients for both the physical and emotional challenges of surgery [20, 21]. Implementation of prehabilitation may also incur additional resources such as workforce for supervising exercise programs and nutritional and psychological support. These could be offset, however, by lower postoperative complications and shorter hospital stays. Hence, prehabilitation could be considered a cost-effective approach overall [22].

## Conclusion:

The effectiveness of postoperative outcomes among patients prepared for elective cardiac surgery with prehabilitation has resulted in reduced complications and postoperative results that improve recovery and thereby shortens overall hospital stays. It stands as an invaluable supplement to fundamental care during the period prior to the surgery, contributing to outcomes of patients that improve along with reducing expenses associated directly with healthcare spend related to postoperative complications and stay in recovery time.

## Figures and Tables

**Table 1:** The baseline characteristics of patients

**Characteristic**	**Group A (Pre-habilitation)**	**Group B (Control)**	**p-value**
Age (Mean ± SD)	68.3 ± 7.9	67.8 ± 8.1	0.711
Gender (Male)	28:22:00	30:20:00	0.745
Type of Surgery	CABG (62%), Valve (38%)	CABG (65%), Valve (35%)	0.635

**Table 2:** The postoperative complications

**Complication Type**	**Group A (Prehabilitation)**	**Group B **	**p-value**
Infections	4%	10%	0.045
Respiratory Complications	6%	12%	0.048
Cardiac Events	2%	6%	0.221
Total Complications	12%	28%	0.016

**Table 3:** The Functional Recovery (6-Minute Walk Test, Meters)

**Time Post-Surgery**	**Group A (Prehabilitation)**	**Group B (Control)**	**p-value**
30 Days	360.2 ± 28.4	320.5 ± 35.1	0.005

**Table 4:** The Functional Recovery (Karnofsky Performance Status Scale)

**Time Post-Surgery**	**Group A (Prehabilitation)**	**Group B (Control)**	**p-value**
30 Days	85.5 ± 5.3	78.9 ± 6.2	0.007

**Table 5:** The Length of Hospital Stay (Days)

**Group**	**Mean Length of Stay (Mean ± SD)**	**p-value**
Group A (Prehabilitation)	6.4 ± 1.8	
Group B (Control)	8.2 ± 2.1	<0.001

**Table 6:** The frequency for Readmission Rates within 30 Days

**Group**	**Readmission Rate (%)**	**p-value**
Group A (Prehabilitation)	4%	
Group B (Control)	10%	0.211

**Table 7:** The Patient Satisfaction with Preoperative Care (1-5 Scale)

**Group**	**Satisfaction Score (Mean ± SD)**	**p-value**
Group A (Prehabilitation)	4.8 ± 0.4	
Group B (Control)	3.9 ± 0.7	<0.001

**Table 8:** The Postoperative Pain Scores (Visual Analog Scale)

**Time Post-Surgery**	**Group A (Prehabilitation)**	**Group B (Control)**	**p-value**
24 Hours	3.5 ± 0.8	4.7 ± 1.2	<0.001
48 Hours	2.8 ± 0.7	3.9 ± 1.0	0.002

**Table 9:** The Time to Extubation (Hours)

**Group**	**Mean Time to Extubation (Mean ± SD)**	**p-value**
Group A (Prehabilitation)	7.2 ± 2.1	
Group B (Control)	10.3 ± 3.4	0.003

**Table 10:** Indicates Postoperative ICU Stay (Days)

**Group**	**Mean ICU Stay (Mean ± SD)**	**p-value**
Group A (Prehabilitation)	1.5 ± 0.6	
Group B (Control)	2.3 ± 0.9	0.004
